# Supplementary material for: Characterisation of a Novel Insect-Specific Virus Discovered in Rice Thrips, Haplothrips aculeatus
Source: Insects. 2024 Apr 24;15(5):303. doi: 10.3390/insects15050303 (PMC11122063; doi:10.3390/insects15050303)
Supplement: Supplementary file 1 [file insects-15-00303-s001.zip › insects-2948781-supplementary Figure S1.pdf]

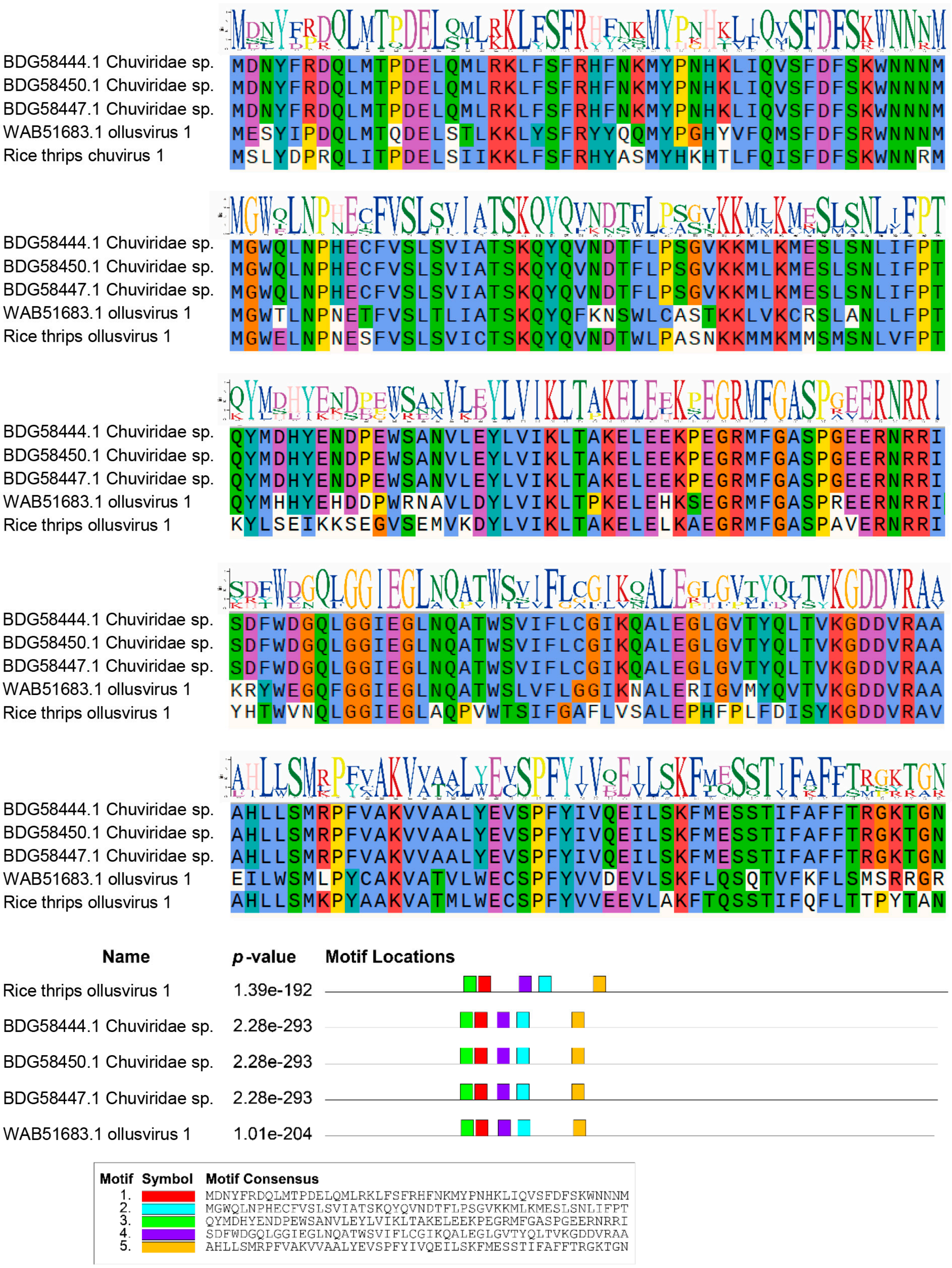

BDG58444.1 Chuviridae sp. MDNYFRDQLMTPDELQMLRKLFSFRHFNKMYPNHKLIQVSFDFSKWNNNM
BDG58450.1 Chuviridae sp. MDNYFRDQLMTPDELQMLRKLFSFRHFNKMYPNHKLIQVSFDFSKWNNNM
BDG58447.1 Chuviridae sp. MDNYFRDQLMTPDELQMLRKLFSFRHFNKMYPNHKLIQVSFDFSKWNNNM
WAB51683.1 ollusvirus 1 MESYIPDQLMTQDELSTLKKLYSFRYYQQMYPGHYVFQMSFDFSRWNNNM
Rice thrips chuvirus 1 MSLYDPRQLITPDELSIIKKLFSFRHYASMYHKHTLFQISFDFSKWNNRM
BDG58444.1 Chuviridae sp. MGWQLNPHECFVSLSVIATSKQYQVNDTFLPSGVKKMLKMESLSNLIFPT
BDG58450.1 Chuviridae sp. MGWQLNPHECFVSLSVIATSKQYQVNDTFLPSGVKKMLKMESLSNLIFPT
BDG58447.1 Chuviridae sp. MGWQLNPHECFVSLSVIATSKQYQVNDTFLPSGVKKMLKMESLSNLIFPT
WAB51683.1 ollusvirus 1 MGWTLNPNETFVSLTLIATSKQYQFKNSWLCASTKKLVKCRSLANLLFPT
Rice thrips ollusvirus 1 MGWELNPNESFVSLSVICTSKQYQVNDTWLPASNKKMMKMMSMSNLVFPT
BDG58444.1 Chuviridae sp. QYMDHYENDPEWSANVLEYLVIKLTAKELEEKPEGRMFGASPGEERNRRI
BDG58450.1 Chuviridae sp. QYMDHYENDPEWSANVLEYLVIKLTAKELEEKPEGRMFGASPGEERNRRI
BDG58447.1 Chuviridae sp. QYMDHYENDPEWSANVLEYLVIKLTAKELEEKPEGRMFGASPGEERNRRI
WAB51683.1 ollusvirus 1 QYMHHYEHDDPWRNAVLDYLVIKLTPKELEHKSEGRMFGASPREERNRRI
Rice thrips ollusvirus 1 KYLSEIKKSEGVSEMVKDYLVIKLTAKELELKAEGRMFGASPAVERNRRI
BDG58444.1 Chuviridae sp. SDFWDGQLGGIEGLNQATWSVIFLCGIKQALEGLGVTYQLTVKGDDVRAA
BDG58450.1 Chuviridae sp. SDFWDGQLGGIEGLNQATWSVIFLCGIKQALEGLGVTYQLTVKGDDVRAA
BDG58447.1 Chuviridae sp. SDFWDGQLGGIEGLNQATWSVIFLCGIKQALEGLGVTYQLTVKGDDVRAA
WAB51683.1 ollusvirus 1 KRYWEGQFGGIEGLNQATWSLVFLGGIKNALERIGVMYQVTVKGDDVRAA
Rice thrips ollusvirus 1 YHTWVNQLGGIEGLAQPVWTSIFGAFLVSALEPHFPLFDISYKGDDVRAV
BDG58444.1 Chuviridae sp. AHLLSMRPFVAKVVAALYEVSPFYIVQEILSKFMESSTIFAFFTRGKTGN
BDG58450.1 Chuviridae sp. AHLLSMRPFVAKVVAALYEVSPFYIVQEILSKFMESSTIFAFFTRGKTGN
BDG58447.1 Chuviridae sp. AHLLSMRPFVAKVVAALYEVSPFYIVQEILSKFMESSTIFAFFTRGKTGN
WAB51683.1 ollusvirus 1 EILWSMLPYCAKVATVLWECSPFYVVDEVLSKFLQSQTVFKFLSMSRRGR
Rice thrips ollusvirus 1 AHLLSMKPYAAKVATMLWECSPFYVVEEVLAKFTQSSTIFQFLTTPYTAN
Name
p-value
Motif Locations
Rice thrips ollusvirus 1 1.39e-192
BDG58444.1 Chuviridae sp. 2.28e-293
BDG58450.1 Chuviridae sp. 2.28e-293
BDG58447.1 Chuviridae sp. 2.28e-293
WAB51683.1 ollusvirus 1 1.01e-204
Motif Symbol Motif Consensus
1. MDNYFRDQLMTPDELQMLRKLFSFRHFNKMYPNHKLIQVSFDFSKWNNNM
2. MGWQLNPHECFVSLSVIATSKQYQVNDTFLPSGVKKMLKMESLSNLIFPT
3. QYMDHYENDPEWSANVLEYLVIKLTAKELEEKPEGRMFGASPGEERNRRI
4. SDFWDGQLGGIEGLNQATWSVIFLCGIKQALEGLGVTYQLTVKGDDVRAA
5. AHLLSMRPFVAKVVAALYEVSPFYIVQEILSKFMESSTIFAFFTRGKTGN
