# Supplementary material for: Characterisation of a Novel Insect-Specific Virus Discovered in Rice Thrips, Haplothrips aculeatus
Source: Insects. 2024 Apr 24;15(5):303. doi: 10.3390/insects15050303 (PMC11122063; doi:10.3390/insects15050303)
Supplement: Supplementary file 1 [file insects-15-00303-s001.zip › insects-2948781-supplementary File S1.pdf]

The COI sequence of *Haplothrips aculeatus*

>ATTTAGATTACGATCCACTAATAGTATTGTTAAAGCTCCAGCTAATACTGGTAACGATAA
TAATAATAGTAGGGAAGTAACTACAATAGATCAAATAAACAATCTAAAATATTCTATTTTT
ATATTTGATATATTAAAAGTAGTTGAAATAAAATTAATTGAACCCAGTAAGGATGAAACC
CCTGCAATATGTAAAGAAATAATGGTTATATCCACAGAAATCCCAACTCTTAAAGACGAT
AAAGGAGGATAAATTGTTCATCCAGTTCCTGAACCTCCTTCCAAAATTATTCTAAATAA
AAGTAAAAATAAAGAAGGAGGTAACAACCAGAATCTTATATTATTTATACGTGGGTAAC
ATATATCTGGGCTCCCCAGTATAATTGGAACTAACCAATTTCCAAACCCTCCTATTATAAT
AGGTATAATTGTGAAAAAAATTATTATAAAGGCATGATTAGTTACAATAACATTATAAGA
TTGACCTGACAAAAAAATATTTCCTGGCTGTATTAGTTCTAAACGAATAATTAAACTTAT
AAATAATCCACAAATTCCAGATCAAAATCCAAAAATAAAATATAATAT
