# Supplementary material for: Characterisation of a Novel Insect-Specific Virus Discovered in Rice Thrips, Haplothrips aculeatus
Source: Insects. 2024 Apr 24;15(5):303. doi: 10.3390/insects15050303 (PMC11122063; doi:10.3390/insects15050303)
Supplement: Supplementary file 1 [file insects-15-00303-s001.zip › insects-2948781-supplementary File S2.pdf]

Three ORFs amino acid sequence of RTOV1

>L protein

MAKSDPPDWREDLTNAFVKQRPRYYRRGRAPKRYVTDSRAFMVDEKFDTMLDLYPYAT
WQQELAEIMTWDAEFINEWSSVISRDCMHELQILGVTPQDICSATLTPCIIAKMVNLALKR
NIYRSTTSKVSDKTLREKACLASLEAVLHQYDHMSSAVTPRIMANFHTKPREKIVEKFKCL
PAEVEILGSVVEDCMRVMSEIKMLHKYVRIKDGRARARFRLHRRLENPWMRLDSLGVRL
LLTRDVSILEVEGRTYLVNHDLILMMINKLVELWSLMLYCHFQDGTNQDEGIYTLQKDFIV
HITDLVLRSGKFGDSTATLTQANKGFLYCKSLEGLFVAECIYRHDFIKGWDNQTLGKTLWK
ALKEEGLPIASEWRKSVLVKMFEKYTMPQIAEVIGTVKIAGHPSIEVEKGLDKLYERTHEL
LEIDSDAVQKTIGVLTRELFMNFHRVHGHYPLCELRADLHGAIRRTIIERLGPTHPVALAAF
KNTDALEWSKVKLLKNAEFDPVDNQLQILKDKALGVTRSKILQVIFGTMPTHEETLTMKT
ENRRALIHFLLNDHYSSEFHKYLSEIKKSEGVSEMVKDYLVIKLTAKELELKAEGRMFGAS
PAVERNRRIVNDKNVMDMMSLYDPRQLITPDELSIIKKLFSFRHYASMYHKHTLFQISFDFS
KWNNRMRKESIDVPAQEFYDRFYGESYIGRTMHMFNNTLYYYARISKNITKRNLTLLNDV
SEDLFQLALASLEKTTKKVQDSELRKTLHKQGQELLAAVQANPSIQNWVTLLERCNIWIG
KYHTWVNQLGGIEGLAQPVWTSIFGAFLVSALEPHFPLFDISYKGDDVRAVIAVPDEAVKN
KGHDGVRQQIMDVMRGLCDQMGWELNPNESFVSLSVICTSKQYQVNDTWLPASNKKM
MKMMSMSNLVFPTLPDLIGSIYSTAHSACAQTTVALPAYCAATYCAAREMLREFGEMSVD
ADGLTALLLWPQILGGPGPLPLQTFMIRGENDMLSAIVALYTHVFQTGSPGLSHRLRSILQV
PMKKTPDKAMLLADPYSVPISAPEQPQAVLKRMMKTSMAKWVRNKDIRSLLSAQGLAD
KTKYVAHLLSMKPYAAKVATMLWECSPFYVVEEVLAKFTQSSTIFQFLTTPYTANEDRAR
ARRAAYRALDKMLDAAQRCLVFWSKIVSNRFIKAQSMLGIPMTHWLSTTTCPTQLTMMI
REKAWNREIIGLTYPSLVVQNMVGSIDELRMLHPNWRLDNIVNNIYVQLHNASSQTDTDS
YHYRAVKGNVPWLGAYTRNDLADVDYRFRIRSTTLSKLKKLFELWKCGESLGPSFQQSV
KVHIQALTSYPLKEALALVPEVGGGHLAHRGVIASTSRVTMNNARPNIMQGVRYESEGLSI
LKGDQTDYSLNYAARQYISILLATWPLQFSLALPEDYPQLLHAYLHWDLAHPDTYRPCPY
CLNDIGDVMITFMLRCPTEITKYRKLTLVGTSKIELNELKRCVNEVVAGRIRRTATQMYLDP
ANPYVQEACAFAVVRKFRLQGQTIQDVHIMNRAQRIPGPQLSELFAATSGYRPAAGLSINL
LRALPVTLLYKSVVMEVLEALLQSLPDLDFDSTIVFDFIAPHWNPLYQFFQTLIHAALLQN
LNLGSTITITQEDWRLSPLQWRAGDNTNAQNAALTFCRHVYPIFRRWIKGEELLPRLIISVI
QPDPQTVTQILHNKVSSLAHALARKLIRRSNTSAKQGILNYFRLETHNEIEAYKVRVDQAE
YVQEIRDLCDQILDDILIGGGIGLMAETLSTNLLHLACNVTLDDWVDAGNLTHIDDLPALQ
TSDPVDIMSLKIGEPFPPSDTALVSGTLIEDIGRRCDKWVLRFALWFSYHLSQTSLVMWEGS
MSDIVQSMLETHKAWLSPFSTIRLCLVTAEDARRVFKHRFDELAAELLAYYDEQTEWQRL
PEHDWARLPNRNVCQLTAVQHTWTQNMHMPHPVLIGSVPSLDWDVLSPILVYLPEGDPL
RGIRLDPREFTRCIGHLNTSASKLTLLLDLTGYVPPEDIIGHYGIMCLADGLAGQTRYCLTH
YTQAIVVYNSLLLNPITGQPVQDASITELPLEIISGGLPREAIGRLKYKGLYPGDLSLPEVIQ
VLCAYSAELHEVSLITFDADRNWSSEPKDYARIIFNIMWLCRRVLTPDGLVIVKLLPTADQS
TMQVLVYWLILFEHAHIIRCPVTRSHSWEIYLIGRGVRPVPEQDIIDLREGTRVLPANPNLE
GLVCEALQPIFDGVRSYIIDGFSNAPHPFGAYRRMLRQQAQPLALQQLVSGFHIEYPQRLT

HLCGWLDELRSRCIVAQESLQSLFRTMNAPNQRPLRLLGRRRQPLASGERLLTSYFTKLCT
MAFVREAVSSYNVGQTYPSLDSLENHLKNAQAHLLESCMEELIDQLLTNPTINVSNMHYT
IQGGCLLLLDDQRKENITTLLRYQAQRWMRIIGTWSYISYVHSENPQLFESLHRHWIESPV
LRCCHEAMIETHTNDRHYVPPPFSPDIRLTPEEAILPEGVPYFR

>N protein

MSRLTLAQRLGACRARQGIPLVAGLSTMTMQRMAVAKPLCPQAFGDISQEVPDVRYTWY
SRIACTGLYVRHMGQINDWNDEQLTSNLIAELGVTWVYDAPDTTAPAINEARQKLIRVIRQ
SITQEDIDTVLHYYVENLYKPEYLGLTEPITVGTMTQQASLQFTAFHKHMLNAVPIHLFQN
PAHHVFRDAILLSGGVEDVALDDPGMGAAAGAGNANAVNQNPLANDTTVADDWANQV
QDWDFQTILNFCHILGIADLNQTMSTAFGAAATLIAVLCKGESVTQNWVVRRAAAVGVIH
GAMDLNDKFNEAVVVSIAKAYKRDNIPLDDLLKYLITTYFSTTGLDTRPYAWMVEQAALS
HITPAIAFAHSIQSMHWANLSYLHTLIPRTQFVNFIHLTLLMYCNPYGGILNPPIPSAHYADV
AYIGLALEKKLDARTTYNQYRGQPRDTIKTRRELDAIVQTMVDQSQSIMDDERTLAKIFT
QLLNVRGTEDGDYCYIPVDNNAALQNVAYEGYVGPVPQLGDQDGNQAAMVAGAIIDNE
REEEIRMATRADWPAKKRLLPRNFARLNRSGMAQAILNNEPQLMKDVRTCCEVLYSISKQ
TSVFNEYTMNAANTQLRVTVTKQLNQELIDIQRRNEIPNAGLCLAPAEHKIITVNIAPAND
DITADSIFNHGDILVTYAIWNNQDVAVHPGYQASVNLPYGFRPAHHQPQQAPPAGVPPNNA
ANAANAALNAAVGGGAGQQLQAAGQVGGNNNGPQNNEGIILEQMDGLGEDLFPVNPQ
NAAGGGGEEEQQEEEQPENEQDDGQQ

>G protein

MILENQSVKLLALFRHIAPRCSKEHIQMMLGAWVTTSVVFMLWVLLSHSPWACAQDPPK
HQLVDDGMNTMIKNETLMFGELGLVLTKIPSVYAVYGSWNYHISLPIPCITGVELGDFNHN
FKNYDNSLTSLFEQLNRTEIIIDPGLKNSIRHLEATQRIAQTSFRNILDSLMNIKQDMETGW
GGIFEPLCDEDIHPLPNQRVTAKGLLATSPAPDHNTNGTSTTPWSSIPVTTERPTTPPVHPAS
ATTSRAKNAAHTGTQTKSGRLVAENMRVDPAMELRTGALLRGKLMSHTAKSKSYKTPAT
PVLPAVPDPNEVLIDGVSSPSNPSLKPESPDVVETFNVADHTSMSWSPATPPIRWTTPLPISST
AAPPASTTSPSFSTTLRASTVKPPETTTSGATGVHGSATTLSNGQSTTVSAEIDSFTGATNPP
NSNDTMSMIQELVEAMGRSKRSIYQIRSVSEPIQQDGTTVSNPPYQQRMHNTHVIHNHNY
EGLFKGSGSFVQVMGGVVNLAPNSGEDPGKVTLLGTPAAEPPPSAERSSPVEALNKEEYN
RLTNQYAAYCSRVPLEDVENCVAPMRRTTYCTDQCKQDFVVHARSHLPEVETMKLDADR
YTAWDDATLNLTYTVLRSVAGPHLTNDVLEDIVEVSNKMHRGRRSNFFAHLWGACSTEER
DAMAAKIESLSTQVGAVHDFTTESIRNMDSLVSAKLAAVDDVVLRMSRNFNDIATQHNNR
LDELRSALLNHTIIVTLIHQLTTMNLYLMSQLKSNELHVQNMEIKMRSLQNTLKTGTISSTL
VTADEIRRATLTISQNLPDGYMLAPVTTLADYYKAPMAKMMLYKSKIYLQLQFDILPSYQ
SYATYQVTALPVAVGSRWFHLRDLPEVFSIHKEAKEPFAWSIMSQAIYKKGMDHPLHTLK
DGMTTGRDWGANCLAKLLSHNPSYNISCVLAEANPNVAAVTERTYIAQVGLNMWAISTN
TTHKVLLHCLDTMSTPKQVIIEHVTTMKIPGQCSINGPAFRLIPYNAALGSTTFTREVYFPY
QLVWGNVSVVPWVPPSLRPSKWVSQVPSVEDLDSLIGNHTLHINNLMIDYKAKQAKTSA

KFNATVEGAKLREMLASMSMNFASWASWVLSAVLILAVIGLIIYCVCVRSNPAVRGASLLG
SIPLQRVAAEVANGTSIVVVNATTTTTIAPPLSNASLIQQIGKVLDHTMSTIQSSFWTPYLFH
TLLWLTGIGLMSFYFYCHVKCLQRVKRRLLVATNWTPRTMNVIHHEGEVKLRLWFIISVK
HLWRKDNITTEAAIQASTLVGNWSDWTCEFNSSQPSFIKAQTFKRWQQVFRIKVSWGSLC
LKSSRYPVLDTCAELPDYLEVKASEILAQCHDVMPWTWGSVEFLNICRVEVIPSQAINLFT
NYGS
