# Supplementary material for: Characterisation of a Novel Insect-Specific Virus Discovered in Rice Thrips, Haplothrips aculeatus
Source: Insects. 2024 Apr 24;15(5):303. doi: 10.3390/insects15050303 (PMC11122063; doi:10.3390/insects15050303)
Supplement: Supplementary file 1 [file insects-15-00303-s001.zip › insects-2948781-supplementary File S3.pdf]

The full genome sequence of RTOV1

>TTCATATACCATTACGTCAAGGGCATGCGATGCTTAGCCAGCAATACGACATGGGGTA
GGAAACAACGGATAGATTTTAAAAAACTACAAAAAGAAAGATAATTCTACACGCATAG
AGGCCAAATAGCTGAAAAACTAAAAGTCACCTGGCCACATCAGTCCGGTACCTCAAAC
CGCGACTCCGCGCCTTTTAAGATTCACATATCTTGCAAATATAGGTCATTCAACGAAAAT
AGGGCACTCCCTCCGGGAGTATAGCCTCTTCTGGGGTCAGCCTTATGTCTGGACTAAAG
GGTGGCGGTACATAGTGCCTGTCATTTGTGTGAGTTTCAATCATGGCTTCATGACAACA
CCTAAGGACTGGTGATTCAATCCAATGTCGGTGTAAAGACTCAAATAGTTGTGGATTTT
CACTATGAACATATGAGATGTAGGACCAAGTTCCTATGATGCGCATCCATCGCTGAGCT
TGATAGCGAAGTAAGGTGGTGATATTCTCCTTGCGTTGATCATCGAGGAGCAAAAGACA
TCCACCCTGAATTGTATAATGCATGTTGGAGACATTGATGGTTGGATTAGTCAACAATTG
ATCTATCAATTCCTCCATACAGGATTCTAATAAGTGTGCTTGAGCGTTTTTCAAATGATT
TTCTAAACTATCCAGGCTAGGGTATGTCTGACCTACATTGTAGCTGCTCACTGCCTCTCT
CACAAAGGCCATTGTGCATAATTTTGTGAAGTATGAGGTCAAGAGCCTTTCACCAGATG
CTAAGGGTTGCCTTCTACGGCCCAACAGCCGTAGGGGTCGTTGGTTGGGAGCATTCAT
TGTGCGGAATAGACTTTGCAAGGATTCTTGTGCGACAATACACCTCGACCGCAGTTCG
TCAAGCCATCCACATAGATGTGTCAGTCGCTGTGGGTACTCTATATGGAACCCTGAAAC
CAACTGTTGCAGAGCAAGCGGTTGTGCTTGTTGGCGTAGCATTCGTCTGTAGGCACCA
AATGGATGTGGTGCATTGCTAAAGCCGTCAATGATATAGGATCTGACCCCGTCAAATAT
GGGTTGAAGGGCCTCACACACCAACCCCTCTAAATTGGGATTTGCTGGTAGTACACGG
GTCCCCTCTCGTAAGTCAATGATATCCTGTTCAGGTACAGGGCGCACGCCCCGCCCTAT
CAAGTATATCTCCCATGAGTGACTTCTAGTTACCGGACACCGAATAATGTGTGCATGCTC
AAAGAGTATCAACCAGTAAACTAGAACTTGCATGGTACTTTGATCTGCTGTGGGCAAC
AATTTGACTATCACTAGGCCATCAGGAGTTAGAACTCGACGACACAACCACATGATATT
GAAGATGATCCTAGCATAATCTTTTGGTTCACTGGACCAGTTCCTATCTGCATCAAATGT
TATCAACGACACTTCATGTAACTCTGCACTATATGCGCATAACACCTGAATTACCTCGGG
TAATGAAAGATCCCCAGGGTACAAGCCCTTGTACTTCAGTCTCCCTATGGCTTCTCTGG
GCAGTCCTCCAGAGATGATTTCTAATGGTAGCTCTGTTATGCTGGCGTCCTGGACAGGT
TGACCCGTTATCGGATTGAGTAAGAGACTATTATAAACCACAATAGCCTGTGTGTAATGT
GTGAGGCAGTACCGTGTTTGACCTGCAAGACCATCTGCAAGACACATGATGCCGTAAT
GCCCGATTATGTCTTCTGGTGGCACATAACCGGTGAGATCTAACAATAGCGTCAATTTG
GAGGCTGATGTATTGAGATGGCCTATGCACCGCGTAAACTCTCGTGGGTCCAGTCGAAT
GCCACGTAAAGGATCACCTTCTGGAAGATATACCAATATGGGGGATAAGACATCCCAGT
CAAGTGATGGCACACTACCGATGAGAACTGGGTGTGGCATATGCATGTTCTGAGTCCA
AGTGTGTTGAACAGCAGTGAGCTGACAAACATTTCGGTTTGGTAGGCGTGCCCAATCA
TGTTCTGGCAACCTTTGCCATTCTGTTTGTTCATCATAATATGCTAGTAGCTCAGCTGCT
AGTTCATCAAATCTGTGTTTAAACACACGACGAGCATCTTCAGCTGTCACCAAGCATAA
TCGTATGGTAGAAAAGGGTGACAGCCATGCCTTGTGAGTCTCTAGCATGCTTTGCACAA
TGTCAGACATTGAGCCCTCCCACATTACTAAGGAAGTCTGGGACAGGTGGTAACTAAA
CCACAATGCGAACCTCAGAACCCACTTATCACACCTACGGCCAATGTCTTCGATCAGG
GTTCCACTCACCAATGCGGTGTCTGAAGGTGGGAAGGGTTCCCCTATCTTCAGACTCAT

TATGTCGACAGGATCCGAGGTCTGTAACGCTGGGAGGTCATCTATATGTGTCAGGTTAC
CAGCATCAACCCAATCGTCAAGAGTAACATTACATGCTAGATGCAGCAGATTAGTGGAC
AGAGTCTCTGCCATAAGCCCTATCCCCCCTCCGATCAATATGTCGTCCAGGATTTGATCA
CACAAATCGCGAATCTCCTGTACGTACTCTGCCTGATCAACCCGAACTTTGTATGCTTCT
ATCTCGTTGTGTGTCTCCAACCGAAAATAGTTGAGAATTCCCTGCTTAGCTGATGTATTG
GATCTCCGTATGAGCTTTCGAGCAAGTGCGTGAGCAAGTGAACTCACCTTGTTGTGGA
GGATTTGTGTCACTGTTTGTGGATCAGGCTGGATCACGGAGATGATCAATCGCGGCAGT
AATTCTTCACCTTTGATCCAGCGCCTGAATATTGGGTACACGTGGCGACAGAAAGTCA
AAGCAGCATTCTGGGCATTGGTGTTATCACCAGCTCTCCATTGCAGTGGACTCAGCCGC
CAGTCTTCCTGTGTTATAGTGATGGTAGATCCTAGGTTCAAGTTTTGCAGCAAAGCTGC
GTGGATGAGTGTCTGGAAGAATTGGTACAACGGATTCCAATGTGGCGCTATAAAGTCA
AACACAATTGTGCTGTCAAAATCCAAGTCAGGAAGTGACTGAAGCAGAGCTTCCAGC
ACCTCCATGACTACACTCTTGTACAACAAAGTGACCGGTAATGCCCTCAGTAGATTGAT
ACTTAGCCCTGCTGCAGGCCTGTAACCTGATGTAGCAGCAAATAATTCCGACAATTGAG
GTCCTGGGATCCTCTGAGCTCTATTCATTATATGGACATCCTGAATGGTTTGTCCTTGCA
ATCTAAACTTACGCACTACTGCAAAAGCACACGCCTCTTGCACGTACGGATTGGCAGG
ATCAAGATACATCTGTGTGGCTGTTCGTCTTATCCGACCTGCTACCACTTCGTTCACACA
CCTCTTTAACTCATTAAGTTCTATCTTGGAAGTACCCACCAATGTAAGTTTCCGGTACTT
AGTGATTTcTGTTGGACAACGTAACATGAATGTTATCATAACATCCCCAATGTCGTTCAG
GCAGTAAGGACAAGGACGGTACGTGTCAGGATGTGCCAAATCcCAATGCAGGTATGCA
TGAAGGAGTTGCGGATAGTCTTCTGGCAGCGCGAGGCTAAACTGTAAGGGCCAAGTA
GCTAATAAGATTGAGATATACTGTCGAGCTGCATAATTGAGGCTGTAATCTGTCTGATCA
CCTTTCAATATACTTAAGCCTTCACTTTCATAACGCACACCCTGCATGATGTTTGGTCTA
GCATTGTTCATGGTCACGCGTGAGGTGGATGCAATCACACCACGGTGAGCCAAATGGC
CTCCACCCACCTCTGGGACTAAGGCTAGAGCTTCTTTCAATGGGTAGGATGTCAAGGC
CTGAATGTGGACTTTCACTGATTGTTGGAAACTCGGGCCGAGACTCTCACCACATTTCC
ATAGCTCAAAGAGCTTTTTCAACTTTGATAGTGTTGTAGATCGTATCCTGAACCTATAAT
CTACATCTGCTAAATCATTGCGGGTGTAAGCTCCTAACCATGGCACATTCCCTTTGACC
GCTCGATAATGATACGAGTCAGTGTCGGTTTGTGAGCTTGCATTGTGAAGTTGTACATA
GATGTTGTTCACAATGTTGTCTAGACGCCAATTGGGATGTAGCATCCTTAATTCATCAAT
ACTACCCACCATATTCTGAACTACCAAACTCGGGTATGTGAGGCCTATGATTTCTCGGTT
CCAAGCTTTTTCACGAATCATCATGGTCAACTGAGTAGGACATGTGGTAGTTGATAACC
AGTGTGTCATTGGGATCCCTAACATAGATTGAGCTTTTATGAACCTATTTGAGACAATCT
TTGACCAAAAAACCAGGCAACGTTGTGCTGCATCGAGCATCTTATCCAGTGCTCTGTA
CGCTGCCCTCCTGGCGCGGGCTCGATCCTCATTTGCAGTATATGGAGTGGTCAAGAACT
GGAAAATTGTGCTACTCTGAGTGAATTTAGCTAACACCTCTTCCACCACATAGAATGGG
CTACACTCCCACAACATTGTTGCCACTTTAGCTGCATAAGGTTTCATGGACAGAAGGTG
GGCTACATATTTGGTTTTATCTGCCAAACCTTGTGCTGATAACAAGGATCTGATGTCCTT
ATTTCGGACCCATTTTGCCATACTAGTCTTCATCATTCTTTTCAGCACAGCTTGTGGCTG
TTCCGGCGCTGAAATAGGTACCGAGTAGGGATCTGCTAATAGCATTGCCTTGTCTGGGG
TCTTCTTCATTGGGACCTGTAAAATTGATCTTAGTCTGTGGCTGAGCCCAGGACTTCCT

GTCTGGAAGACATGTGTGTATAATGCAACAATTGCTGATAACATGTCATTCTCGCCTCTT
ATCATAAATGTTTGTAGAGGGAGGGGCCCTGGACCTCCTAATATCTGTGGCCACAGGAG
CAAAGCAGTTAGGCCATCAGCATCCACTGACATCTCACCGAACTCTCGTAACATTTCTC
GAGCAGCACAATATGTCGCGGCACAGTACGCTGGTAATGCAACCGTGGTTTGGGCACA
AGCAGAATGAGCAGTTGAGTAGATACTCCCAATCAGGTCAGGCAATGTAGGGAAAACC
AAGTTAGACATGGACATCATCTTCATCATCTTCTTGTTGGATGCTGGGAGCCATGTATCA
TTGACCTGGTATTGTTTACTAGTGCAGATCACGCTTAGGGATACAAAAGATTCATTGGG
GTTCAATTCCCAACCCATTTGATCACAGAGTCCACGCATCACATCCATAATCTGCTGTCT
GACACCGTCATGTCCTTTGTTTTTACTGCCTCATCTGGCACTGCAATGACAGCTCGCA
CATCATCTCCTTTGTATGAGATGTCGAATAACGGAAAATGTGGCTCCAGCGCGCTGACA
AGGAAGGCTCCAAATATTGATGTCCAGACTGGTTGTGCCAACCCTTCGATGCCCCCTAA
TTGATTTACCCATGTATGGTATTTTCCGATCCAGATATTACATCTCTCTAACAAAGTGACC
CAGTTCTGGATACTAGGATTAGCTTGCACAGCAGCTAGTAGCTCTTGTCCTTGTTTATGT
AATGTCTTACGTAACTCGCTATCCTGGACTTTTTTGGTCGTTTTTTCAAGTGATGCCAAA
GCCAGTTGGAACAAGTCCTCTGATACATCGTTTAGGAGTGTCAGATTCCTCTTGGTTAT
ATTCTTACTGATACGGGCATAATAATACAATGTGTTATTGAACATGTGCATTGTACGACC
GATATACGATTCACCATAAAAACGATCATAAAACTCTTGAGCAGGAACGTCAATAGACT
CCTTCCTCATCCGATTATTCCACTTCGAGAAATCAAAACTGATCTGGAACAGGGTATGC
TTGTGGTACATTGATGCGTAATGTCTAAAGGAGAATAATTTCTTGATGATGCTCAACTCA
TCTGGTGTGATCAACTGCCGCGGGTCATACAACGACATCATGTCCATCACATTCTTGTC
ATTTACAATTCTCCTGTTTCTTTCCACAGCCGGACTAGCTCCAAACATGCGCCCTTCTG
CTTTCAGCTCTAACTCCTTGGCAGTCAGTTTAATCACCAAGTAATCTTTCACCATCTCAG
AAACCCCCTCACTCTTCTTGATCTCACTCAGATACTTATGAAATTCAGATGAATAATGGT
CATTGAGCAAGAAATGTATGAGTGCACGTCGATTTTCAGTTTTCATTGTGAGGGTTTCT
TCATGAGTGGGCATTGTGCCAAAGATAACTTGTAATATCTTACTACGTGTCACCCCCAA
AGCTTTGTCTTTCAAGATTTGCAACTGGTTGTCTACCGGATCGAATTCCGCATTCTTCA
ACAATTTTACTTTCGACCATTCGAGGGCATCTGTGTTCTTGAATGCTGCGAGAGCCACC
GGGTGTGTGGGGCCTAATCTCTCAATGATGGTTCGACGAATAGCGCCATGTAGGTCCGC
TCTCAACTCACATAGTGGATAATGACCATGGACTCGATGAAAATTCATGAATAGCTCAC
GTGTGAGGACTCCTATAGTCTTCTGCACTGCATCGGAGTCAATTTCCAACAATTCGTGT
GTCCTTTCATAAAGCTTATCAAGTCCCTTCTCAACCTCAATACTAGGATGTCCAGCAATT
TTAACTGTGCCAATCACTTCTGCTATCTGAGGCATTGTGTATTTCTCAAACATCTTGACC
AGCACAGACTTCCTCCATTCTGAAGCAATTGGAAGTCCTTCCTCCTTTAATGCCTTCCA
TAATGTTTTCCCCAATGTTTGGTTATCCCATCCTTTGATAAAATCATGTCGGTAAATGCAT
TCTGCTACAAACAAGCCTTCCAGTGATTTACAGTATAGGAACCCCTTGTTAGCCTGTGT
CAAAGTGGCTGTGGAATCACCGAATTTCCCACTACGAAGGACCAAGTCAGTAATGTGC
ACAATAAAATCCTTTTGCAGAGTGTAGATGCCCTCATCCTGATTAGTGCCATCTTGGAA
ATGACAATACAGCATTAGAGACCATAATTCCACTAGCTTGTTTATCATCATCAGTATGAG
GTCATGGTTGACGAGGTATGTCCGACCCTCTACCTCCAATATGCTCACATCTCTCGTCAA
TAGTAATCGGACACCTAAACTATCAAGTCTCATCCATGGATTTTCCAATCTACGATGAAG
GCGGAACCTTGCTCGTGCTCTACCATCCTTAATCCTCACATACTTATGCAACATTTTTAT

CTCACTCATCACTCTCATACAATCTTCCACCACAGAACCTAGAATTTCCACTTCGGCTG
GGAGGCATTTGAACTTCTCTACAATTTTCTCTCTAGGTTTAGTATGGAAGTTCGCCATAA
TCCTCGGAGTGACCGCAGAAGACATGTGATCGTATTGGTGCAAGACTGCCTCGAGAGA
TGCTAGACAGGCCTTCTCTCTCAGAGTTTTATCAGACACTTTTGATGTAGTCGAACGAT
AGATGTTACGCTTAAGAGCAAGATTGACCATTTTCGCTATGATACAGGGTGTGAGTGTG
GCTGAGCAAATATCTTGCGGAGTAACACCTAAGATCTGTAACTCGTGCATACAGTCTCG
ACTTATTACAGAAGACCATTCATTTATGAACTCTGCATCCCAGGTCATGATCTCCGCTAA
CTCTTGCTGCCACGTGGCGTAGGGATATAGGTCTAGCATTGTATCGAATTTCTCATCTAC
CATAAACGCTCTAGAATCTGTCACATATCGTTTGGGGGCTCTGCCTCGTCTGTAGTACC
GTGGTCGTTGTTTGACAAAAGCATTAGTCAAATCTTCCCGCCAATCAGGCGGATCGGA
CTTTGCCATCCTCTAACAGCAAAGTTTAAGCGATCAAGGCAATACCTGAACTCAACAA
AGATTGAGTTTTAAACCATGTGAATAATGTTTTTGTCGCCTTCACAAGTTTATTTTTCAT
TTTTTATTTTCCCTCTCACGCAGTGCAAATCACCAGCACCCCATCACCAAAAGTAAGTC
ACTCTCCATCACCAATAGCTTCCCCACAGCAATAGATTTAAATTCTATCAACAGAGTATA
ATCGGGGTCTACCACCCCGGGCTGCTAAGTACACATATAGCTGATACAAGGGCCATGTG
TTAGCCACGCGTAGTACATCCCCAACAGGATGTTTAGCCATCAACACTTACTGTTGGCC
ATCGTCTTGTTCGTTTTCCGGTTGCTCTTCCTCCTGTTGCTCTTCCTCCCCACCACCTCC
AGCAGCGTTTTGTGGGTTCACAGGGAATAGATCTTCACCGAGACCGTCCATCTGTTCC
AAGATTATGCCCTCATTGTTCTGGGGTCCATTGTTGTTGCCGCCGACCTGTCCCGCTGC
CTGGAGCTGTTGACCTGCCCCTCCACCGACTGCAGCATTGAGAGCAGCATTCGCAGCA
TTAGCTGCGTTGTTTGGTGGTACTCCAGCTGGTGGCGCCTGTTGTGGTTGATGGTGAGC
AGGTCTGAACCCATAGGGTAGGTTCACTGATGCCTGATATCCGGGGTGGACTGCAACG
TCCTGATTGTTCCAGATGGCATATGTCACCAGAATATCCCCATGATTGAAGATGGAATCA
GCAGTGATGTCATCATTAGCAGGTGCAATGTTCACAGTGATGATCTTGTGTTCCGCAGG
AGCTAAGCACAAACCAGCGTTCGGTATTTCATTTCTTCTCTGGATGTCAATCAATTCCT
GGTTCAGCTGCTTGGTTACTGTCACTCGAAGTTGTGTATTCGCGGCATTCATGGTGTAC
TCATTGAATACGGAGGTTTGTTTGCTGATTGAGTACAGGACCTCACAGCACGTTCTCAC
ATCCTTCATGAGTTGGGGCTCATTGTTCAGAATTGCTTGTGCCATCCCGGATCGATTGA
GTCTTGCGAAATTTCGGGGAAGCAATCTTTTCTTGGCTGGCCAATCTGCTCGTGTTGCC
ATCCGAATCTCCTCTTCTCGTTCATTGTCGATGATGGCACCAGCGACCATAGCAGCTTG
GTTACCATCTTGATCTCCCAATTGCGGGACCGGACCGACATAGCCTTCATATGCAACATT
CTGGAGAGCCGCATTGTTATCCACTGGGATGTAGCAATAATCACCATCTTCAGTACCCC
TTACATTGAGCAACTGGGTGAAAATCTTGGCTAAAGTGCGTTCATCATCCATGATTGAT
TGGGATTGATCCACCATCGTCTGGACGATGGCGTCTAGTTCACGGCGGGTTTTTATAGT
GTCTCTTGGCTGCCCTCTGTATTGATTGTAGGTTGTGCGTGCGTCTAGTTTCTTCTCTAA
TGCCAATCCGATATATGCCACATCAGCGTAGTGCGCTGAAGGGATCGGAGGGTTAAGGA
TTCCTCCATACGGGTTGCAGTACATGAGCAAAGTCAGGTGAATGAAATTGACGAACTG
GGTCCTAGGGATAAGTGTGTGCAAGTAGGAGAGATTGGCCCAGTGCATACTTTGGATC
GAGTGAGCAAAAGCAATAGCTGGAGTGATATGACTCAATGCAGCTTGCTCGACCATCC
ATGCATAAGGGCGAGTATCTAGCCCTGTTGTGCTGAAGTAGGTAGTGATGAGGTATTTC
AAGAGGTCATCCAAAGGTATATTGTCCCTCTTGTAAGCCTTAGCAATGGACACAACAAC

CGCTTCATTGAACTTATCATTGAGGTCCATTGCGCCATGGATCACACCTACAGCAGCAG
CCCGCCGGACAACCCAATTTTGAGTGACTGACTCGCCTTTGCACAATACCGCTATCAAT
GTTGCAGCGGCTCCAAAAGCTGTAGACATGGTTTGATTTAAGTCAGCAATACCGAGGA
TATGGCAGAAGTTGAGAATTGTTTGGAAGTCCCAATCCTGCACTTGGTTGGCCCAGTCA
TCAGCCACTGTGGTGTCATTGGCCAATGGGTTCTGATTCACTGCATTAGCATTGCCTGC
GCCTGCAGCTGCGCCCATGCCTGGATCATCGAGAGCAACATCTTCGACACCTCCTGAC
AGCAGGATGGCGTCTCGGAACACATGATGCGCAGGGTTCTGGAAAAGATGGATCGGA
ACTGCATTAAGCATGTGCTTATGAAATGCAGTGAATTGGAGACTCGCCTGCTGGGTCAT
CGTACCCACGGTGATAGGCTCTGTGAGTCCTAGATATTCAGGCTTGTACAAATTTTCTAC
ATAGTAATGCAGCACTGTGTCAATATCCTCTTGAGTGATACTTTGACGAATTACCCGAAT
TAGTTTCTGCCGAGCCTCATTGATTGCCGGAGCTGTGGTGTCAGGGGCATCGTACACCC
ATGTAACACCCAGTTCCGCAATTAGGTTCGAGGTCAGTTGTTCGTCATTCCAGTCATTG
ATTTGACCCATATGACGCACGTATAACCCGGTACAGGCGATTCTACTATACCATGTGTAA
CGCACATCGGGGACCTCTTGCGAGATGTCACCAAATGCTTGGGGACACAGTGGTTTTG
CAACCGCCATGCGTTGCATGGTCATAGTACTCAGGCCGGCCACTAAGGGTATGCCCTGA
CGTGCACGGCACGCTCCTAACCTCTGTGCTAACGTCAAGCGAGACATGATTCAGCACA
ATGCAGTTTGGCTTGTACACGTCAATGTATAACAACGTCCTAACTCAGGATGCAATTAA
CGAATCAAAAGGGGAACAAACAATAGAGTTTTAAACCATGTATTTGTCCTTCGACAATT
GTGTGTTGGCATATTTAGTCTGGTCCAACATATGTGAAAGCCAATGACCCCATGAGAAG
CAAATGATTTTTTGCCTATTATAGCTCAGTTACTTGCAGTTATCCCTGAGCCTGATGTTTA
ATACGTTTCTGGGCACGTCGTGTATCGAACTACTGTAAGTCATGTTTATATATGAGTTAT
GGTGCAATGCCACTGCACATCCCCTTAGTGGAGTCAATAATAGTAGCATCTGTGATCAT
GTTGTCGTCAGAACTGGAAAGCACCTCATAGAGCCCTGGTGCACAACAGTTGGTACTA
CTTATAAGGATCTGATATGTGCCAACACGCATGATTGAGTCAATCACCTCACTTCACATT
ACGCGATGCGGTGATGTCTCGTGCAGTACGCGGGTAATTGGAGGTGGTCACTTGGGGC
CTGGATCTCAGCTCCAAGAGGAGATTCTTCTGCAGGGTCAACATACCCGTTATCTTGTC
CAGCTTTGAGTCGATTGTTTGCAATGTCAGCTCCTGCTTCTGTCCGGGGGATACTGCGG
CTATGGGGGTTTGACGGATAGCACTCATTGATTGCATGTTGCTGGGTGGTGCAGAGGG
AGCGGCTTCACGAGTTTTCATCATGTTGTTCATGTTGGGCCTCATTCAAATGTTGCTAAT
ACTGCAAGCAAGAAACCTGAGAGAAAAATACCAAAGTTTTAAACCACGATAAAGTGT
TTGTTGTCCTTCAACGTGTGGCATTTTTTATTTTTATTTTTTCTGAACACATGGTTAATAA
TATAAGGATAATATCTTTGATTAAATGTCACAACAAGGTGGTCAATCTATGATTCAGTAA
ATTGTATGTCTTTCATTCATGCTACCCTTTACAACAATGCGCACAGTGTTTATCATTTATT
TAATGTCTGAGGACATTGGAGGCATAATATCTGGCTTGCAAATTTAGGAAGGCTAGGTA
GATTAAACCTCTGATAAAGGGATTGAACAGTAGATCAATTAAGGGCTGATTGGTATGCT
ACTTGGCAATGCAACAGACCTGAACCTTGTGTGGGCAGTGTGGTTATTACGTACGCGA
TTAGAAATACACCCCACTTAATCCCTTGTGGGCAACCTTAATCAAATACCCAGTAAATC
CTTGTCATCATATCTGGTGATCATCACATGCTTATTCAGATCATCCATCAATGGACACTCT
TAAGGAGTTATGATTAATAGTTAACTAAGAGGTATGCTATCCATATTATCCCAACTAGTAC
TCAATCTCTTAACCCCTCCGAATATGACACTGGAAGATCTGGTGAGATCGTAAATAGAC
ACTCCGGGGGCCACTTCTAGTCAGGATCCGTAATTGGTGAACAAGTTAATTGCCTGTGA

TGGGATTACCTCCACACGACATATGTTCAAGAATTCAACGCTCCCCCACGTCCATGGCA
TAACATCATGACACTGAGCCAAGATTTCAGATGCCTTCACTTCAAGGTAGTCAGGCAG
CTCAGCACAAGTGTCAAGGACCGGATAACGACTACTCTTCAGACACAGTGAACCCCAT
GATACCTTGATCCGAAAGACTTGCTGCCATCGTTTGAATGTTTGCGCCTTGATGAAGGA
TGGTTGCGAACTATTGAACTCACAGGTCCAGTCCGACCAATTCCCAACCAAGGTTGAG
GCTTGGATAGCCGCCTCTGTGGTAATGTTGTCTTTGCGCCACAGATGCTTCACACTTATT
ATGAACCAAAGTCTTAACTTTACTTCACCTTCATGATGGATTACGTTCATGGTTCTTGGT
GTCCAGTTGGTGGCAACGAGCAAGCGACGCTTCACACGCTGCAAGCATTTGACATGG
CAATAAAAGTAGAAGGACATCAATCCAATTCCGGTTAGCCATAGAAGTGTGTGGAATA
GATAAGGTGTCCAGAAGCTGCTTTGAATAGTGCTCATAGTGTGGTCCAATACTTTCCCA
ATTTGCTGGATGAGACTGGCATTGCTCAACGGGGGGGCAATTGTGGTTGTGGTTGTTG
CATTGACCACCACAATACTAGTCCCATTAGCAACCTCAGCTGCCACTCTCTGTAATGGT
ATTGAACCAAGGAGCGAAGCTCCTCTCACAGCTGGATTAGAGCGTACACACACACAGT
AGATAATAAGACCTATGACTGCCAGGATCAGCACAGCTGACAGAACCCAGCTGGCCCA
AGAGGCAAAATTCATGCTCATACTGGCCAACATTTCGCGCAGTTTAGCACCCTCTACAG
TGGCATTGAACTTTGCTGATGTCTTTGCCTGCTTAGCTTTGTAGTCAATCATCAGATTGT
TAATATGGAGCGTATGATTCCCGATCAGTGAATCTAGGTCTTCCACAGATGGGACTTGT
GATACCCATTTACTTGGTCTTAAGCTGGGAGGCACCCATGGGACAACACTAACATTCCC
CCACACAAGTTGGTAAGGGAAATAAACTTCACGTGTAAATGTTGTGCTTCCCAATGCG
GCATTGTAAGGGATTAATCGGAATGCAGGTCCATTAATGGAGCATTGACCCGGAATTTT
CATGGTGGTCACATGTTCGATGATGACTTGCTTGGGTGTGCTCATAGTATCCAAACAGT
GTAACAAGACCTTGTGTGTAGTGTTTGTGCTTATTGCCCACATATTTAGTCCAACCTGA
GCTATATATGTCCTTTCAGTGACAGCAGCTACATTGGGATTGGCTTCAGCCAGGACACA
GCTGATATTGTAGCTAGGATTGTGGGACAGGAGTTTTGCTAAGCAGTTAGCTCCCCAAT
CTCTACCAGTAGTCATTCCATCTTTCAAAGTGTGCAGTGGGTGATCCATACCCTTCTTGT
AAATTGCCTGACTCATGATTGACCAAGCAAATGGTTCCTTTGCTTCCTTGTGTATCGAG
AATACTTCCGGCAAGTCGCGGAGATGGAACCACCGACTCCCCACTGCTACAGGGAGTG
CTGTCACTTGGTAAGTTGCATAACTCTGATAGGAGGGCAAGATGTCAAATTGCAATTGG
AGATAGATTTTGGATTTATAAAGCATCATCTTCGCCATAGGTGCTTTGTAGTAATCAGCT
AGGGTTGTGACAGGGGCAAGCATATAACCATCTGGCAGATTCTGAGAGATAGTTAAAG
TAGCTCGTCGAATCTCATCAGCTGTGACAAGTGTCGAGGAAATGGTGCCAGTCTTGAG
AGTGTTCTGTAGAGAACGCATTTTGATTTCCATATTTTGTACATGTAGTTCATTAGACTT
CAGCTGAGACATTAGGTATAGGTTCATAGTCGTTAGTTGGTGTATTAGAGTGACTATGAT
AGTATGGTTGAGCAGTGCTGATCGCAGCTCATCTAGTCGATTGTTGTGTTGTGTAGCGA
TGTCATTGAAGTTTCTTGACATCCGCAGCACCACATCATCGACTGCTGCTAACTTGGCT
GATACAAGTGAATCCATATTCCGTATTGACTCAGTTGTGAAATCATGAACAGCGCCCAC
TTGAGTTGATAACGATTCAATTTTGGCAGCCATTGCATCTCGTTCTTCAGTGCTACAAG
CTCCCCATAAGTGTGCAAAGAAATTGCTGCGTCGTCCTCGGTGCATCTTGTTAGATACC
TCGACTATATCTTCTAGCACGTCATTGGTGAGGTGAGGGCCAGCAACTGACCGGAGCA
CTGTATATGTCAGATTCAAGGTTGCATCATCCCATGCGGTATACCGATCCGCATCCAATT
TCATCGTCTCAACTTCAGGGAGGTGACTCCGTGCATGGACGACAAAATCTTGTTTGCAT

TGGTCAGTGCAATAGGTTGTACGTCGCATCGGCGCTACACAGTTCTCAACATCTTCAAG
CGGCACACGGCTACAATAAGCTGCATACTGATTGGTCAACCTGTTGTATTCCTCCTTGT
TCAAAGCTTCTACTGGGCTGGATCTTTCAGCACTGGGAGGAGGTTCTGCTGCCGGGGT
CCCTAAGAGTGTCACCTTCCCTGGGTCTTCACCAGAGTTAGGGGCTAAATTCACCACC
CCTCCCATAACCTGGACAAATGACCCGCTACCTTTAAATAGGCCTTCATAATTGTGATTG
TGGATGACATGAGTGTTATGCATGCGTTGTTGGTAAGGCGGGTTGGACACTGTTGTGCC
ATCCTGCTGGATTGGCTCAGACACTGACCGGATTTGGTAAATTGATCTCTTGGAACGAC
CCATCGCCTCAACCAACTCTTGGATCATGGACATGGTATCATTGCTGTTAGGCGGATTA
GTTGCTCCAGTGAAGGAGTCAATTTCTGCAGACACGGTGGTAGACTGTCCATTGGACA
AAGTTGTTGCACTACCGTGAACTCCTGTCGCCCCAGATGTGGTGGTCTCCGGAGGTTT
CACTGTACTAGCTCGTAGTGTGGTGGAAAAGCTAGGACTGGTAGTGGATGCAGGGGGA
GCAGCTGTACTGCTGATTGGGAGAGGAGTGGTCCATCTGATTGGGGGGGTAGCTGGAG
ACCAACTCATACTAGTGTGATCAGCAACATTGAATGTTTCCACAACATCAGGCGATTCT
GGCTTCAGACTTGGATTGGACGGTGAGCTTACACCATCTATGAGTACCTCATTGGGATC
TGGGACTGCTGGGAGTACTGGGGTTGCTGGTGTCTTGTAAGATTTCGATTTGGCTGTAT
GTGACATCAACTTGCCTCGTAACAAGGCTCCGGTGCGCAATTCCATGGCAGGGTCGAC
CCTCATATTCTCTGCCACCAATCTACCTGATTTTGTCTGGGTCCCTGTGTGAGCCGCATT
TTTGGCTCTACTGGTTGTTGCTGATGCCGGGTGGACTGGAGGTGTAGTTGGTCTCTCGG
TAGTCACTGGGATACTTGACCAGGGTGTGGTTGAAGTACCATTCGTGTTGTGGTCAGG
AGCTGGGCTAGTGGCGAGTAAACCTTTTGCTGTTACCCGTTGGTTGGGCAAAGGATGG
ATATCTTCGTCACACAGGGGTTCGAATATTCCGCCCCACCCTGTTTCCATATCCTGTTTG
ATGTTCATGAGTGAATCTAGGATGTTTCTAAAACTAGTCTGTGCGATGCGTTGTGTGGC
CTCCAGATGACGGATAGAATTCTTGAGTCCCGGATCAATTATGATTTCCGTGCGATTGA
GTTGTTCGAAAAGACTGGTGAGGCTGTTATCATAATTCTTGAAATTATGATTGAAATCA
CCCAGCTCGACCCCAGTTATGCAGGGGATAGGTAGGGATATGTGATAATTCCAAGAACC
ATAAACAGCATACACAGATGGTATCTTTGTGAGCACAAGACCAAGTTCCCCAAACATC
AGGGTCTCATTCTTGATCATGGTGTTCATACCATCGTCAACTAATTGGTGTTTGGGCGGA
TCTTGGGCGCAGGCCCATGGGCTATGACTGAGCAAAACCCAGAGCATGAACACCACA
GAAGTGGTGACCCACGCTCCAAGCATCATTTGGATGTGTTCCTTACTGCACCTGGGAG
CAATATGACGAAATAAGGCCAACAATTTTACAGATTGGTTTTCAAGGATCATGAGCCTT
GAAGATAAACCTCTGTTTTTTTGTTGTCCTTCAATATTTGTCCGTTTGTTCA
