# Supplementary material for: Characterisation of a Novel Insect-Specific Virus Discovered in Rice Thrips, Haplothrips aculeatus
Source: Insects. 2024 Apr 24;15(5):303. doi: 10.3390/insects15050303 (PMC11122063; doi:10.3390/insects15050303)
Supplement: Supplementary file 1 [file insects-15-00303-s001.zip › insects-2948781-supplementary Table S1.pdf]

Supplementary Table S1. Primers used in this study.

Primer	Position	Sequence (5' to 3')	Purpose
F1	1-21	TTCATATACCATTACGTCAAG	Amplification of RTOV1 viral genome
R1	1687-1706	CGGCATCATGTGTCTTGCAG	
F2	1638-1657	GCCTGTGTGTAATGTGTGAG	
R2	3325-3344	GGTAGCAGGTCGGATAAGAC	
F3	3279-3298	TGCACGTACGGATTGGCAGG	
R3	4962-4981	TGTGCCGCGACATATTGTGC	
F4	4916-4935	AGCATCCACTGACATCTCAC	
R4	6622-6641	AGCAGAAGTGATTGGCACAG	
F5	6570-6589	AGCTTATCAAGTCCCTTCTC	
R5	8287-8306	GGACCCCAGAACAATGAGGG	
F6	8233-8252	GGGTTCACAGGGAATAGATC	
R6	9956-9975	TGTCGAAGATGTTGCTCTCG	
F7	9906-9925	CACTGCATTAGCATTGCCTG	
R7	11628-11647	TTTAATCTACCTAGCCTTCC	

F8	11584-11603	ATGTCTGAGGACATTGGAGG	
R8	13309-13328	TGGGCAATAAGCACAAACAC	
F9	13253-13272	TGACTTGCTTGGGTGTGCTC	
R9	14975-14994	CAATGGACAGTCTACCACCG	
F10	14926-14945	CTGTTAGGCGGATTAGTTGC	
R10	16262-16282	TGAACAAACGGACAAATATTG	
5RACE-R1	4495-5023	CATTCTGCTTGTGCCCAAACCACGGTTGC	5’RACE of full RTOV1 genome sequence
5RACE-R2	3272-3301	GATCCTGCCAATCCGTACGTGCAAGAGGC	
3RACE-F1	16119-16146	GGTGACCCACGCTCCAAGCATCATTTGG	3’RACE of full RTOV1 genome sequence
