# Supplementary material for: Characterisation of a Novel Insect-Specific Virus Discovered in Rice Thrips, Haplothrips aculeatus
Source: Insects. 2024 Apr 24;15(5):303. doi: 10.3390/insects15050303 (PMC11122063; doi:10.3390/insects15050303)
Supplement: Supplementary file 1 [file insects-15-00303-s001.zip › insects-2948781-supplementary Table S2.pdf]

Supplementary Table S2. The BLAST results of RTOV1 compare to the NCBI NT and NR database.

Blast program	Query sequence	Top hit viruses	Query coverage	E-value	Identify	Accession
BlastX	Full genome of RTOV1	Chuviridae sp.	38%	0	32.76%	BDG58450.1
		Chuviridae sp.	38%	0	32.83%	BDG58447.1
		Chuviridae sp.	38%	0	32.76%	BDG58444.1
BlastP	ORF1 protein of RTOV1	Beetle aliusvirus	59%	6e-22	21.74%	WPR17560.1
		Bat faecal associated chuvirus 3	53%	2e-20	21.74%	UVF62170.1
		Bat faecal associated chuvirus 2	53%	8e-18	22.04%	UVF62166.1
	ORF3 protein of RTOV1	ollusvirus 1	65%	8e-19	21.61%	WAB51682.1
		Hubei coleoptera virus 3	46%	3e-18	21.45%	YP_009336865.1
		Culverton virus	30%	3e-15	22.48%	YP_010798360.1
	ORF4 protein of RTOV1	Chuviridae sp.	87%	0	32.09%	BDG58444.1
		Chuviridae sp.	87%	0	32.09%	BDG58450.1
		Chuviridae sp.	87%	0	32.15%	BDG58447.1
